# Supplementary material for: A pilot study of stable isotope fractionation in Bombyx mori rearing
Source: Sci Rep. 2023 Apr 24;13:6643. doi: 10.1038/s41598-023-33790-z (PMC10126144; doi:10.1038/s41598-023-33790-z)
Supplement: Supplementary file 1 — Supplementary Tables. [file 41598_2023_33790_MOESM1_ESM.docx]

**Table S1** Mean values of stable carbon isotope ratio in the samples

| Sample | Isotope ratio/‰(n=5) | | |
| --- | --- | --- | --- |
|  | JSHY | HYJS | HK3 |
| Mulberry leaf | -29.00±0.08 | | |
| Newly-hatched silkworm | -29.26±0.11 | -29.23±0.04 | -29.30±0.04 |
| Silkworm excrement | -28.62±0.09 | -28.59±0.05 | -28.50±0.06 |
| Silkworm at the fifth instar | -29.24±0.03 | -29.20±0.07 | -29.26±0.07 |
| Silkworm pupa | -30.75±0.08 | -31.75±0.12 | -32.74±0.05 |
| Silkworm cocoon | -25.75±0.05 | -26.75±0.07 | -25.44±0.10 |

**Table S2.** Mean values of stable nitrogen isotope ratio in the samples

| Sample | Isotope ratio/‰(n=5) | | |
| --- | --- | --- | --- |
|  | JSHY | HYJS | HK3 |
| Mulberry leaf | 1.76±0.17 | | |
| Newly-hatched silkworm | 3.63±0.09 | 3.95±0.18 | 2.67±0.11 |
| Silkworm excrement | 2.26±0.14 | 2.16±0.15 | 2.09±0.17 |
| Silkworm at the fifth instar | 2.52±0.10 | 2.60±0.13 | 2.51±0.09 |
| Silkworm pupa | 2.57±0.15 | 2.31±0.17 | 2.01±0.18 |
| Silkworm cocoon | 1.16±0.11 | 2.10±0.16 | 2.43±0.16 |

**Table S3.** Mean values of stable hydrogen isotope ratio in the samples

| Sample | Isotope ratio/‰(n=5) | | |
| --- | --- | --- | --- |
|  | JSHY | HYJS | HK3 |
| Mulberry leaf | -76.72±1.89 | | |
| Newly-hatched silkworm | -99.02±1.32 | -97.90±3.02 | -108.22±2.89 |
| Silkworm excrement | -70.67±2.22 | -68.39±1.62 | -69.36±2.32 |
| Silkworm at the fifth instar | -99.67±2.17 | -97.71±1.98 | -101.29±2.46 |
| Silkworm pupa | -99.52±1.89 | -126.06±1.95 | -134.51±2.19 |
| Silkworm cocoon | -77.41±1.64 | -68.35±2.45 | -70.35±1.88 |

**Table S4.** Mean values of stable oxygen isotope ratio in the samples

| Sample | Isotope ratio/‰(n=5) | | |
| --- | --- | --- | --- |
|  | JSHY | HYJS | HK3 |
| Mulberry leaf | 31.04±0.11 | | |
| Newly-hatched silkworm | 25.18±0.16 | 24.73±0.10 | 25.75±0.13 |
| Silkworm excrement | 29.35±0.13 | 29.17±0.13 | 29.38±0.11 |
| Silkworm at the fifth instar | 26.10±0.12 | 26.29±0.15 | 26.06±0.12 |
| Silkworm pupa | 19.86±0.15 | 22.00±0.09 | 22.56±0.10 |
| Silkworm cocoon | 28.23±0.13 | 28.80±0.12 | 28.55±0.14 |

**Table S5-1** Mean values of stable hydrogen, oxygen, carbon, and nitrogen isotope ratio in JSHY under starvation conditions.

| Starvation  Time/h | Isotope ratio/‰(n=5) | | | |
| --- | --- | --- | --- | --- |
|  | δ^2^H | δ^18^O | δ^13^C | δ^15^N |
| 0 | -107.41±1.02 | 26.19±0.12 | -29.68±0.03 | 1.69±0.07 |
| 6 | -106.55±0.89 | 25.78±0.13 | -29.51±0.04 | 1.83±0.06 |
| 12 | -111.93±0.86 | 24.78±0.16 | -29.52±0.04 | 2.03±0.04 |
| 24 | -116.96±0.82 | 25.19±0.11 | -29.5±0.05 | 1.70±0.05 |
| 36 | -117.69±0.74 | 24.67±0.11 | -29.48±0.04 | 2.18±0.06 |
| 48 | -110.09±0.88 | 24.83±0.10 | -29.27±0.04 | 2.16±0.07 |
| 60 | -109.29±0.69 | 23.48±0.13 | -29.12±0.03 | 2.36±0.05 |
| 72 | -102.65±0.82 | 24.2±0.15 | -29.11±0.02 | 2.45±0.08 |

**Table S5-2** Mean values of stable hydrogen, oxygen, carbon, and nitrogen isotope ratio in HYJS under starvation conditions.

| Starvation  Time/h | Isotope ratio/‰(n=5) | | | |
| --- | --- | --- | --- | --- |
|  | δ^2^H | δ^18^O | δ^13^C | δ^15^N |
| 0 | -107.48±0.85 | 26.88±0.09 | -29.71±0.03 | 2.38±0.06 |
| 6 | -108.05±0.74 | 26.47±0.12 | -29.65±0.02 | 2.4±0.07 |
| 12 | -121.87±0.98 | 24.39±0.14 | -29.68±0.04 | 2.25±0.08 |
| 24 | -122.1±1.01 | 24.38±0.14 | -29.42±0.03 | 2.65±0.05 |
| 36 | -117.14±0.75 | 25.56±0.12 | -29.47±0.04 | 2.25±0.05 |
| 48 | -111.18±0.87 | 23.65±0.13 | -29.13±0.01 | 2.13±0.04 |
| 60 | -102.18±0.80 | 24.25±0.10 | -29.18±0.03 | 2.10±0.06 |
| 72 | -106.85±0.76 | 23.88±0.11 | -29.23±0.02 | 1.67±0.07 |

**Table S6-1** Mean values of stable hydrogen, oxygen, carbon, and nitrogen isotope ratio in JSHY before and after starvation

| Sample | Isotope ratio/‰(n=5) | | | |
| --- | --- | --- | --- | --- |
|  | δ^2^H | δ^18^O | δ^13^C | δ^15^N |
| Control | -107.52±1.32 | 26.21±0.15 | -29.65±0.03 | 1.68±0.08 |
| Starved for 72h | -103.83±1.11 | 24.22±0.11 | -29.09±0.04 | 2.52±0.07 |
| Resumed diet for 72h | -85.09±1.15 | 28.59±0.12 | -28.95±0.05 | 2.10±0.06 |

**Table S6-2** Mean values of stable hydrogen, oxygen, carbon, and nitrogen isotope ratio in HYJS before and after starvation

| Sample | Isotope ratio/‰(n=5) | | | |
| --- | --- | --- | --- | --- |
|  | δ^2^H | δ^18^O | δ^13^C | δ^15^N |
| Control | -107.47±1.16 | 26.92±0.09 | -29.71±0.04 | 2.4±0.05 |
| Starved for 72h | -106.99±1.21 | 23.89±0.10 | -29.22±0.06 | 1.68±0.07 |
| Resumed diet for 72h | -95.15±1.27 | 27.31±0.13 | -28.73±0.05 | 1.07±0.06 |
